# Steroid-induced delayed diagnosis of primary vitreoretinal lymphoma with ghost brain tumor: A case report

**DOI:** 10.1097/MD.0000000000029637

**Published:** 2022-07-22

**Authors:** Hui-Chuan Kau, Cheng-Jen Wang, Chieh-Chih Tsai

**Affiliations:** a Department of Ophthalmology, Koo Foundation Sun Yat-Sen Cancer Center, Taipei, Taiwan; b Department of Ophthalmology, School of Medicine, National Yang Ming Chiao Tung University, Hsinchu, Taiwan; c Department of Neurosurgery, Koo Foundation Sun Yat-Sen Cancer Center, Taipei, Taiwan; d Department of Ophthalmology, Taipei Veterans General Hospital, Taipei, Taiwan.

**Keywords:** ghost brain tumor, primary vitreoretinal lymphoma, uveitis masquerade syndrome

## Abstract

Primary vitreoretinal lymphoma is a great masquerader and provides a diagnostic challenge.

It is most frequently misdiagnosed as a chronic uveitis. Steroid treatment for presumed uveitis can interfere with the correct diagnosis of vitreoretinal lymphoma. Herein, we present a case of primary vitreoretinal lymphoma in which the correct diagnosis was delayed by short-term steroids until 2 years later.

**Patient concerns:**

A 45-year-old woman presented with floaters and blurred vision in her right eye for 3 months. An ocular examination revealed dense vitreous cells. Three months later, she developed headache and suicidal ideation after taking a 3-week medication of oral steroid medication from another eye clinic. Brain magnetic resonance imaging revealed a tumor involving the corpus callosum and periventricular region.

**Interventions:**

Vitreous biopsy and repeated brain biopsies were carried out for the patient.

**Diagnosis:**

A brain biopsy was performed for the first time, and a vitreous biopsy was performed when steroid medication was suspended for 20 and 41 days, respectively. Both biopsies were negative for the presence of malignant cells. Follow-up magnetic resonance imaging revealed complete remission of the brain tumor. Two years later, the tumor recurred in the optic chiasm. Diffuse large B-cell lymphoma was confirmed by a second brain biopsy.

**Outcome:**

The patient had complete tumor remission after receiving brain radiation therapy and chemotherapy.

**Lessons:**

Vitreoretinal lymphoma is difficult to diagnose owing to its rarity, masquerading presentation, and steroid-induced apoptosis of lymphoma cells. Physicians should consider vitreoretinal lymphoma as an important differential diagnosis in patients presenting with chronic uveitis and use steroids cautiously before making a definitive diagnosis.

## 1. Introduction

Primary vitreoretinal lymphoma (PVRL), previously known as ocular reticulum cell sarcoma, is a rare and fatal malignancy. Patients with PVRL usually present with blurred vision and floaters and less commonly have red eyes, photophobia, and ocular pain.^[1]^ The most common clinical signs of PVRL are vitreous cells and opacity, which may form clumps and sheets, and infiltrative lesions located deep in the retina.^[2]^ The symptoms and signs of PVRL mimic those of chronic uveitis, which often delays correct diagnosis. Moreover, the empirical use of steroids for uveitis may further interfere with tissue diagnosis.^[3]^ In this report, we present a case of PVRL with subsequent brain involvement. The patient had a definitive diagnosis 2 years later because of the short-term use of corticosteroids, which led to negative results from diagnostic vitrectomy and brain biopsy.

## 2. Case report

A 45-year-old woman presented with floaters and blurred vision in her right eye for 3 months. She had a history of breast cancer with complete remission for 5 years.

On examination, corrected visual acuity was 6/8.6 in the right eye and 6/6 in the left eye. The cornea and lens are clear. Cells or flares were not observed in the anterior chamber. Fundus examination revealed a vitreous opacity with many floating cells. No retinal lesions, including hemorrhage, exudation, or infiltration, were found. Under the suspicion of chronic uveitis, she received 1% prednisolone eye drop treatment and underwent blood tests for autoimmune diseases. However, the vitreous cells persisted, and blood tests for complete blood count, erythrocyte sedimentation rate, antinuclear antibody, rheumatoid factor, human leukocyte antigen B27, and complement 3 and 4 were all within normal limits.

**Figure 1.**
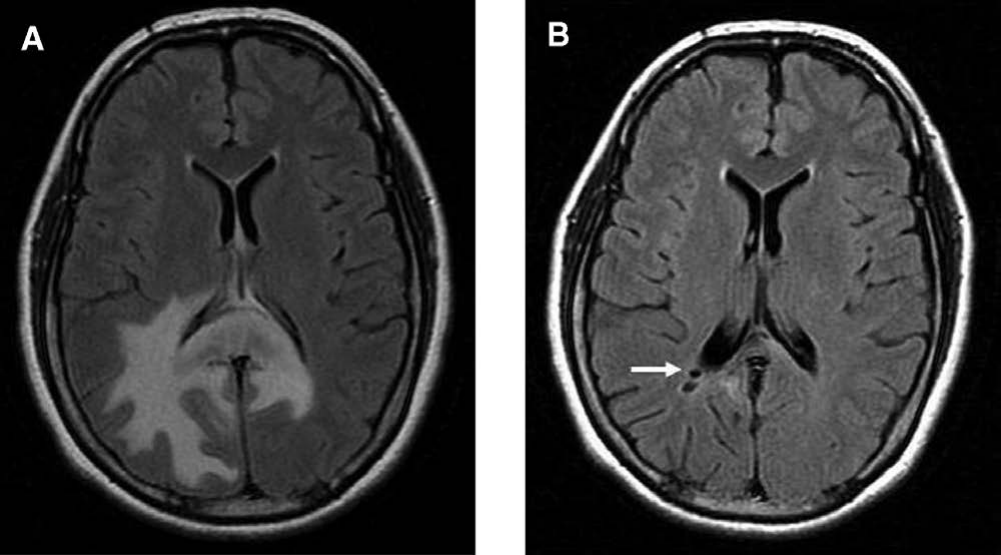
Vanishing tumor in MRI series. (A) Brain MRI showed a contrast-enhanced tumor with perifocal edema involving the corpus callosum and periventricular region. (B) Four months later, the brain tumor totally disappeared. The biopsy tract is visible (white arrow). MRI = magnetic resonance imaging.

The patient did not return visit until 3 months later. She reported severe headache and suicidal ideation for 3 weeks since she had received oral steroid medications of unknown dosage from an eye clinic. The visual acuity in the right eye was 6/8.6, and vitreous cells remained. Based on the persistence of vitreous cells and the recent-onset headache, PVRL with brain involvement was suspected. Brain magnetic resonance imaging (MRI) performed after she stopped all medications for 9 days showed a lobulated contrast-enhanced tumor with perifocal edema involving the corpus callosum and periventricular region (Fig. 1A). Subsequently, she underwent a navigation-guided brain biopsy 11 days after the MRI. However, histological examination revealed only benign nervous tissue with focal lymphohistiocytic infiltrates. Postoperative MRI revealed regressed and less-enhanced brain tumors. As there was no definite diagnosis from the brain tissue, she underwent diagnostic vitrectomy 3 weeks later. However, only a few atypical lymphoid cells were observed in this specimen. She underwent regular follow-up with repeated brain MRI examinations 4 months later, which revealed that the brain tumor had completely disappeared (Fig. 1B).

**Figure 2.**
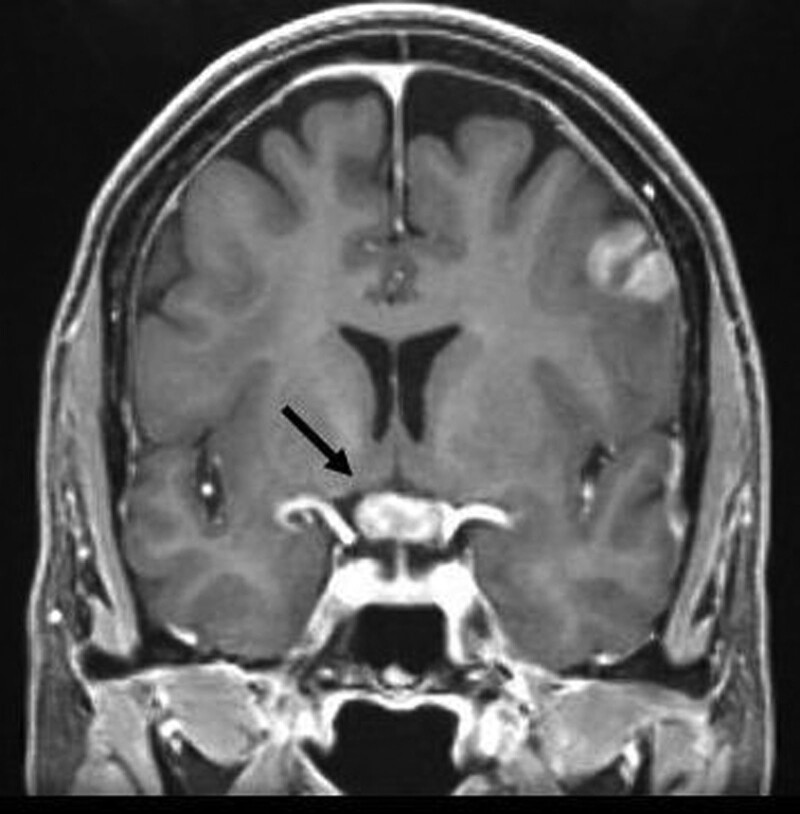
Two years later, multiple brain tumors were found and one of them involved the optic chiasm (black arrow).

**Figure 3.**
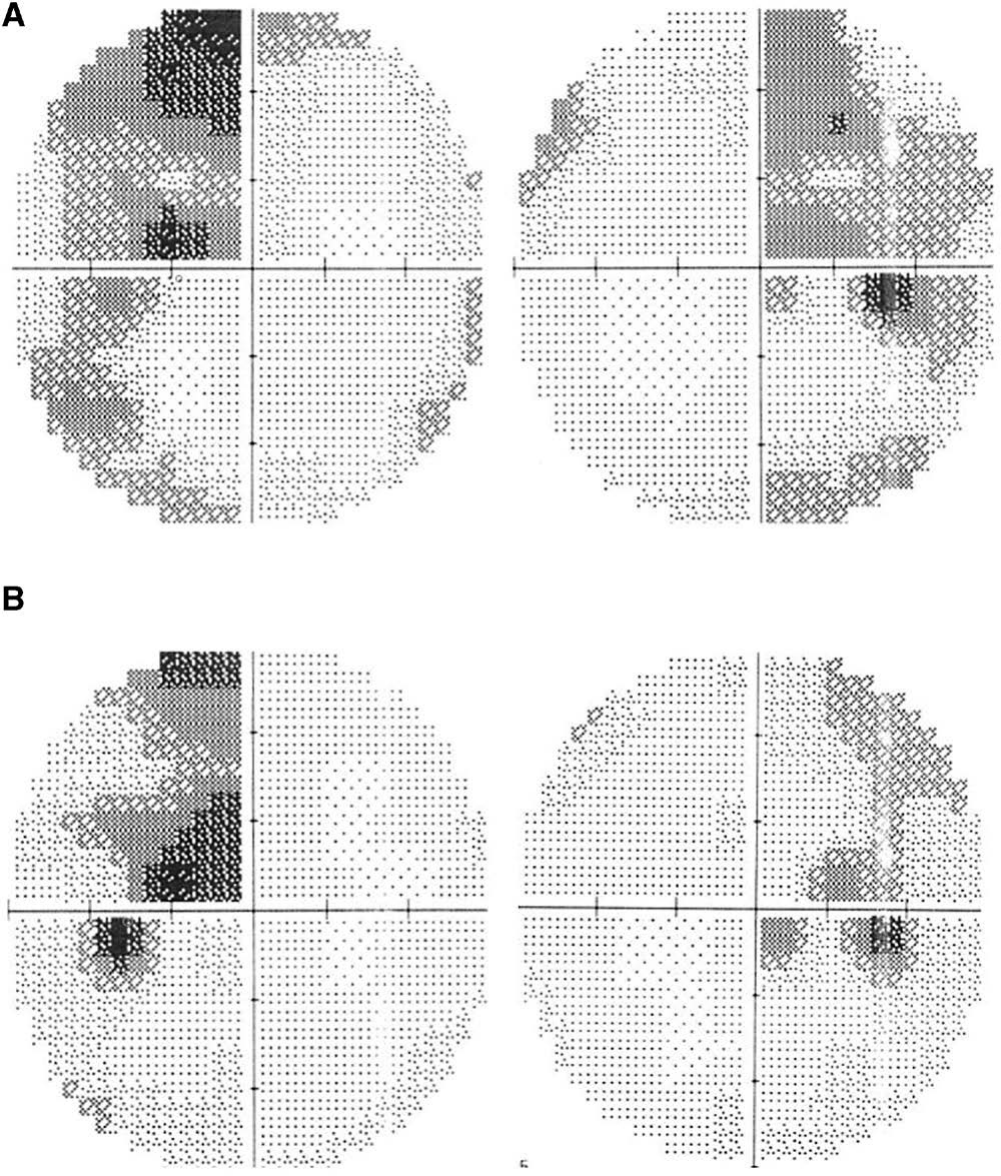
Visual field defect. (A) Visual field test performed when brain radiation and chemotherapy were just completed revealed bitemporal hemianopia. (B) Three months later, the follow-up visual field test showed improvement.

Two years later, the patient experienced rapid visual deterioration in both eyes within 2 weeks. She could see only the fingers at about 1 m away in both eyes. The anterior chamber and vitreous humor were free of cells. Brain MRI revealed multiple tumors, one of which involved the optic chiasm (Fig. 2). Brain biopsy revealed diffuse large B-cell lymphoma. The patient subsequently received brain radiation therapy and chemotherapy, with complete tumor remission. Her vision improved to 6/7.5 in the eyes. The visual field test showed bitemporal hemianopia, which improved during the follow-up (Fig. 3A, B).

## 3. Discussion

PVRL is a subset of primary central nervous system lymphoma (PCNSL). Over 50% of patients with PVRL eventually develop brain lymphoma and 15% to 20% of patients with PCNSL present with intraocular lymphoma.^[4]^

PVRL is difficult to diagnose early in clinical practice. The reasons for this are as follows. First, PVRL is a great masquerader. The clinical signs of PVRL mimic a wide variety of ocular diseases, including infectious uveitis (e.g., tuberculosis, toxoplasmosis, and acute retinal necrosis), noninfectious uveitis (e.g., intermediate or posterior uveitis, multifocal choroiditis, Behcet disease, sarcoidosis), and metastatic cancer.^[2]^ Second, PVRL has a low incidence of 0.047 cases per 100,000 people per year.^[5]^ It is a rare disease compared with chronic uveitis. Clinical suspicion of PVRL will not be the first priority in the long list of differential diagnoses if there are no accompanying symptoms of the central nervous system. Third, the biopsy results of diagnostic vitrectomy have a high false-negative rate. This is mainly due to poor sample volume, paucicellular specimens, and necrotic lymphoma cells.^[2,6]^ Mishandling vitreous specimens and prior treatment with steroids can lower the diagnostic yield. Repeated biopsy procedures are often required to establish a definitive diagnosis. Delayed diagnosis of PVRL often leads to the development of brain lymphoma.^[7]^

Steroids have a direct apoptotic effect on lymphoma cells. They should be avoided before biopsy when PVRL or PCNSL is suspected. However, steroids often have been used earlier for treating presumed uveitis or for reducing brain edema. Discontinuation of steroids for at least 2 weeks before biopsy has been suggested in the literature.^[8–11]^ In the present case, a brain biopsy was performed 20 days after steroid suspension. The result was negative for malignant cells even though there was radiological persistence of the brain lesion. Likewise, the vitreous cells persisted, and diagnostic vitrectomy was performed 43 days after cessation of oral and topical steroids. However, no lymphoma cells were observed in vitreous specimens. The lymphocytic effects of steroids may be profound and long-lasting.^[12,13]^ In the present case, the brain lesions and vitreous cells gradually disappeared and lasted over 2 years due to the prior 3-week steroid treatment only.

In conclusion, PVRL is a rare but important differential diagnosis in patients with chronic uveitis. Steroids should be avoided before tissue diagnosis when lymphoma is suspected.

## Author contributions

Conceptualization, H.-C.K.; methodology, C.-C.T. and H.-C.K.; validation, C.-J.W., H.-C.K., and C.-C.T.; formal analysis, C.-C.T. and H.-C.K.; investigation, C.-J.W., H.-C.K., and C.-C.T.; resources, C.-J.W. and H.-C.K.; writing, H.-C.K. and C.-C.T. All authors have read and agreed to the published version of the manuscript.
